# Feasibility and Cost-Effectiveness of Daycare Anterior Cruciate Ligament Reconstruction - A Retrospective Case Series

**DOI:** 10.5704/MOJ.2411.001

**Published:** 2024-11

**Authors:** J Khan, R Ali, S Fahad, F Mariam, N Baloch

**Affiliations:** Department of Surgery, Aga Khan University, Karachi, Pakistan

**Keywords:** ACL reconstruction, day care surgery, cost effectiveness, anterior cruciate ligament

## Abstract

**Introduction:**

Multiple reviews of the applicability, cost-effectiveness, and safety of daycare reconstruction of anterior cruciate ligament have been published in French, American, and British setups, but have not been described in our population.

**Materials and methods:**

In this study, 25 patients who underwent Anterior Cruciate Ligament Reconstruction (ACLR) as a daycare surgery in our setup were assessed retrospectively. Post-operatively patients were reviewed for pain, complications, conversion from daycare to inpatients, readmission within two weeks post ACLR and cost-effectiveness.

**Results:**

None of the patients required readmission within two weeks post-operatively, two patients were admitted on request from Surgical Day Care (SDC) to inpatient postoperatively, two patients developed urinary retention. Daycare ACLR was also cost-effective, as shown by cost analysis a reduction of cost by 26.9 %.

**Conclusion:**

Daycare ACLR is safe, feasible, and cost-effective treatment modality for young patients and can provide a substantial cost saving.

## Introduction

The most common ligamentous injury to the knee joint is an anterior cruciate ligament (ACL) tear^1^. The subsequent knee instability is successively managed by ACL reconstruction (ACLR) and patients have a greater chance to return to their previous physical activity status^2,3^. Reconstructions of anterior cruciate ligament using a hamstring graft or the central third of the patellar tendon have been conventionally done on an inpatient basis with a post-operative hospital stay of 1-2 days^4-6^. The stay is rationalised based on a need to provide post-operative pain control, antibiotics, early mobilisation, and close observation for potential complications. With less invasive procedures, evolution in arthroscopy, and the advent of better analgesia we are now able to do ACLR on a daycare or outpatients’ basis, this is widespread in developed countries^7,8^, but is not as frequently practiced in developing countries. The incidence of daycare surgery for ACLR in the USA has increased significantly from 57.3% in 1997 to 95.1% in 2006^9^. The rate of daycare ACLR procedures increased by 300% between 1994 and 2007, while inpatient procedures have been significantly decreased by 70.6% between 1994 and 2006^10^. In our setup, we are doing ACLR on a daycare basis since 2019.

The aims for the transition of ACLR surgery, from in-patient to daycare surgery are cost reduction, the need to save inpatient bed and resources (which can be utilised for other patients), and most importantly their feasibility and equal outcomes. Other benefits are decreased vulnerability and exposure to hospital-acquired infections^10^. In addition, patients are allowed to recover in a safe, private, and relaxed home environment. Lefevre N *et al* showed the feasibility of a short one-day hospital stay^11^. Patients with short-stay admission were significantly more satisfied and had less pain than those who had gone through conventional hospitalisation of 2-3 days. For health insurance entities a saving indirect costs ranging from -25% to -68% and enhancement in the yield and productivity of the healthcare setting has been reported in the literature. In 2019, Barbier *et al* reviewed the economic effect of daycare surgery on the French healthcare system and reported a 34% cost reduction of ACLR on a daycare basis compared with inpatient settings^12^.

We retrospectively evaluated 25 consecutive daycare ACLR patients to look at its feasibility and cost-effectiveness.

## Materials and Methods

A retrospective consecutive case series study was conducted at our institution from January 2017 to Feb 2020. Institutional ethical review committee exemption was taken. Data of patients were collected from medical record files. SPSS version 20 was used for data entry and analysis. Files were reviewed for age, gender, co-morbidities, duration of surgery, tourniquet time, and early outcomes [including pain score {graded from 0-10 on visual analogue score}, conversion from daycare to inpatient, the reason for conversion to inpatient, and re-admission with the reason (within a week)]. The cost was calculated by reviewing surgical daycare final bills from the billing department of our hospital and daycare patients' bills were compared to those who were converted to inpatients from SDC.

Patient with complete ACL tear diagnosed on clinical examination and MRI with age ranging from 15-50 years with no serious health condition requiring in-hospital supervision after the operation (ASA I, II) and no known hypersensitivity to NSAIDs, or any known bleeding disorder or active peptic ulcer disease were included in the study. Patients having the previous ACLR, multi-ligament injury, incomplete record/ files, or patients lost to follow-up were excluded from the study.

All patients were electively admitted from the clinic. Preoperative anaesthesia assessment was done, and the patient's ASA level was recorded. Patients were preferably kept first on list when available and admitted to daycare before 8:00 am on an empty stomach to start the surgery at 8:00 am. As a protocol SDC unit of our hospital do not accepts admissions after 4pm. So, if the first slot was not available due to list sharing or other cases on list then all effort was done to start the case before 4pm. All patients whose surgery finished after 6pm (closing time for SDC), were kept in recovery for extended time until ready for discharge and were discharged as per protocol from the recovery room. General anaesthesia and a single dose of cefazolin as prophylactic antibiotics were given to all patients, tourniquet applied before prepping with draping, the tourniquet was inflated once all markings were done and surgeon is ready for incision within a minute, and pressure of tourniquet kept at 300mmHg for a maximum of 2 hours for hemostasis. In case of prolonged surgery, the tourniquet was deflated for 10 minutes and then reinflated. All procedures were done arthroscopically, and grafts used for ACLR were quadrupled hamstring (semitendinosus), bone-patella tendon-bone graft (BPTB graft). The graft was either fixed with endo buttons, metallic screws, bioabsorbable screws, a combination of screws and endo buttons. Meniscus injury was either repaired with all inside or inside out techniques if repairable or partial meniscectomy was done. The wound was closed with a 3/0 ethelone. No drain was placed in any case. In all cases, a tourniquet was released after dressings.

Post-operatively all patients remained in recovery for 30-60 minutes and were then shifted to surgical daycare from where they were discharged home. The pain was assessed as soon as the patient recovered from anaesthesia using a visual analogue score (VAS) (from 0 no pain to 10 maximum pain). The patient's discharge was not approved if there was significant pain (VAS >3), pain requiring narcotics, or a postoperative complication requiring observation or surgical treatment. It was also assured that the patient is tolerating diet with no nausea, vomiting, the patient has passed urine, is vitally stable, fully conscious, oriented with no bleeding from the wound, no soakage of dressing, and no undue swelling with soft compartments of the leg. Patients were discharged home with a removable knee immobiliser brace.

Oral Paracetamol 1gm Q6H and NSAIDs combination (Diclofenac Sodium 50mg Q8H) was systematically prescribed for 5 days. In the case of residual pain oral tramadol 50mg also advised on as required basis. Patients were advised to keep his/her lower limb elevated at home along with icing to minimise soft tissue swelling and provide analgesia by regional cooling of the knee. Weight bearing status was decided based on choice of graft and additional meniscal procedures performed. All patients were reviewed in the consulting clinic on the first available clinic of the operating surgeon (ranging from first post day to no later than 3rd post op day) and at one week after the first follow-up for an overall assessment regarding wound, swelling, and pain.

## Results

A total of 25 patients were included in the analysis out of which 21 (84%) were males and 4 (16%) were females with a mean age of 25.64 years +/- 7.6 SD. The mean BMI of our patients was 25.69 +/- 4.4 SD. Mean tourniquet time was 140.36 minutes +/- 22 SD. Mean surgery time was 170.72 minutes +/- 42.5 SD. Mechanism of injury in 18 patients (72%) were sports injuries, the most common sports associated with an ACL injury in our study was football, followed by cricket. After sports the most common reason for ACL injury was direct trauma to the knee and ground-level fall, which accounted for 24% of the cases (6 patients). Only one patient (4%) had a history of RTA. Right ACL tear was observed in 13 (52%) patients and left in 12 (48%) patients. In 4 cases (16%) ACL injury was associated with a medial meniscus injury, 7 cases (28%) were associated with a lateral meniscus injury and 2 cases (8%) were associated with both medial and lateral meniscus injuries. While in 12 cases (48%) there was isolated ACL injury (Table I). In 7 cases (28% of the meniscus injuries) partial meniscectomy was performed while in 6 cases (24%) meniscus was repaired, of these 6 cases 5 menisci were repaired using all inside techniques and one was repaired using inside-out technique (Table II). For ACL reconstruction we used Quadrupled hamstring graft in 13 cases (52%) and BPTB graft in 12 cases (48%).

**Table I:** Patient demographics and characteristics of ACL injuries.

S.No	Variable	Mean+SD/Frequency (%)
1 Age (in years)		25.64+7.5
2 BMI (kg/m^2^)		25.69+4.4
3 Gender	Male	21 (84%)
	Female	4 (16%)
4 Mechanism of injury	Sports	18 (72%)
	Trauma other than sports	7 (38%)
5 Injuries	Isolated/pure ACL injury	12 (48%)
	ACL + lateral meniscus injury	7 (28%)
	ACL+ medial meniscus injury	4 (16%)
	ACL+ medial and lateral menisci injury	2 (8%)

**Table II:** Treatment done during surgery.

S.No	Treatment done		Frequency (%)
1 Meniscus injury (n=13)	Partial menisectomy		7 (53.84%)
	Repair	All inside	5 (38.46%)
		Inside out	1 (7.7%)
2 Graft used for ACL (n=25)	BPBT graft		12 (48%)
	Quadruple hamstring (Semitendinosus)		13 (52%)
3 Fixation techniques used for graft fixation (n=25)	Metallic screws Bioabsorbable screws		5 (20%) 7 (28%)
	Metallic+ Bioabsorbable screws		1 (4%)
	Endobuttons		12 (48%)

The graft was fixed using endobuttons in 12 cases (48%), bioabsorbable and metallic screws were used in 7 cases (28%) and 5 cases (20%), respectively. In one case a metallic screw was used on the femoral side and a bioabsorbable on the tibial side. Post-operatively most patients (58%) were having a pain score on VAS <3 while the rest (32%) of patients had a VAS score of >4 which was addressed with Inj. Tramadol 50mg which on reassessment reduced to a VAS score of 2. Two patients developed post-op urinary retention, so in and out catheterisation was done in one case, and in the second case urology was taken on board, and Foley’s catheter was passed and patient was discharged with Foley’s catheter which was removed after one week following consultation with urology team. Mean hospital stay was 12.5 +/- 2.4 hours. One patient presented in the clinic on 5th POD with hematoma and oozing from the wound site of BPTB graft harvesting, which was drained and examined for any bleeder, there was no active bleeder, so a stitch was applied in the clinic. There was no readmission noted within 2 weeks for any complications, while two patients (8%) were converted from daycare to inpatients on their wishes, both were fulfilling the discharge criteria, one of them were operated on late hours and he was from a far-flung area, so he stayed for a day. We also compared the different variables like duration of surgery, tourniquet time, post-op pain, and complications between the two graft techniques. We found no significant difference among the two groups in terms of these variables (Table III). Interestingly we observed a 26.9% reduction in cost among patients who underwent daycare ACL reconstruction as compared to those who converted from daycare to a one-day stay as an inpatient.

**Table III:** Comparison of the variables among two graft techniques.

S.No	Variable		PTBT graft (n=12)	Hamstring Graft (n=13)	Significance p-value
1.	Duration of surgery (mean in minutes)		174.42+52.6	167.31+32.7	0.692
2.	Tourniquet time (mean in minutes)		137.75+20.1	141.85+24.3	0.731
3.	Post-op pain (mean VAS)		3.42+0.9	3.0+1.0	0.308
4.	Post-op complications	Urinary retention	1	1	0.367
		No complication	11	12	

## Discussion

The global surgical practice for elective surgery is swiftly moving towards enhanced recovery after surgery (ERAS) protocols. Similarly, the trend toward doing ACLR on a daycare basis instead of inpatients is growing and its safety, practicality, and cost-effectiveness have been recognised^13^. Losee *et al* reviewed a series of day surgery ACLR using semitendinosus in 135 patients and a patellar tendon graft in 182 patients. No patient required post-operative readmission for pain relief or wound complications^14^. Other complications were comparable to those of inpatient ACLR. Using a daycare surgery practice, a saving of $5900 per patient was achieved. Kao *et al* compared the cost of day surgery vs inpatient ACLR and found an average inpatient cost of $9220 while an average day surgery cost of $3905 (around 57% cost reduction)^15^. Kumar *et al* have discussed the role of day surgery in a prospective study of 20 patients. They had a 0% re-admission rate and reported a 100% success rate in terms of patient satisfaction^16^. This study was conducted in a University Hospital where we have experienced a similar scenario.

To our knowledge, this is the first study in Pakistan evaluating the feasibility of daycare ACLR in Pakistan. Our study supports the fact that ACLR performed using general anaesthesia can be predictably accomplished as a day surgery procedure.

In our study, we did meniscectomy or meniscus repair along with ACLR in 64% of cases, with even prolonged surgery, anaesthesia, and tourniquet time. All these patients successfully recovered. So, we conclude through our study that some complex knee injury which includes ACL tear along with meniscus injury can be easily managed as daycare surgery.

Timing of tourniquet is important in this surgery. The maximum time of tourniquet in most of the cases (58%) was 150 minutes, however in 8 cases (32%) expected timing exceed up to more than 150 minutes. In these cases, we released the tourniquet after 120 minutes and then re-inflated it after 10 minutes, no complications were reported due to increased tourniquet time.

All our patients were comfortable in the recovery area and were able to be transported to their homes with minimum discomfort. No major complication requiring conversion from daycare to inpatients or readmission was noted (within one week) in our study.

Post-operatively family counselling was done to explain the procedure and post-operative care with emphasis on two important aspects, weight bearing status at home for necessary movement and signs where they should visit the emergency department. Standard instructions were to keep the limb in immobiliser, do elevation as needed and some icing over the dressing to provide cooling effect, until seen in clinic on follow-up.

In this study, the sample size was small and future randomised studies are necessary and justified to further evaluate patient satisfaction, cost-effectiveness, and longterm outcome.

## Conclusion

In conclusion, this study has demonstrated that day surgery ACLR can be both a feasible and safe procedure in the appropriately selected patients and can provide a substantial cost saving. It is important that the surgeon and medical staff spend adequate time with the patient and thoroughly discuss the expected course of recovery. Finally, for either patient preference or medical indications, the choice for admission, must be completely taken by the orthopaedist and patient.

## References

[1] Vakti SK, Bethi AR, Mahadvuni D, Rao SM, Katyayani MS. (2021). Study of correlation between MRI and arthroscopic findings in anterior cruciate ligament and meniscal injuries of the knee joint.. Int J Orthop..

[2] Pedersen M, Grindem H, Johnson JL, Engebretsen L, Axe MJ, Snyder-Mackler L (2021). Clinical, Functional, and Physical Activity Outcomes 5 Years Following the Treatment Algorithm of the Delaware-Oslo ACL Cohort Study.. J Bone Joint Surg Am..

[3] Somashekar D, Kumar S, Ponnam RM, Kumar K. (2021). Arthroscopic ACL reconstruction-functional outcome with BPTB graft with interferential screws.. Int J Orthop..

[4] Ajrawat P, Dwyer T, Whelan D, Theodoropoulos J, Murnaghan L, Bhargava M (2021). A comparison of quadriceps tendon autograft with bone-patellar tendon-bone autograft and hamstring tendon autograft for primary anterior cruciate ligament reconstruction: a systematic review and quantitative synthesis.. Clin J Sports Med..

[5] Zhao L, Lu M, Deng M, Xing J, He L, Wang C (2020). Outcome of bone–patellar tendon–bone vs hamstring tendon autograft for anterior cruciate ligament reconstruction: A meta-analysis of randomized controlled trials with a 5-year minimum follow-up.. Medicine (Baltimore)..

[6] Lin KM, Boyle C, Marom N, Marx RG (2020). Graft selection in anterior cruciate ligament reconstruction.. Sports Med Arthrosc Rev..

[7] Valkering KP, van Bergen CJA, Buijze GA, Nagel PHAF, Tuinebreijer WE, Breederveld RS (2015). Pain experience and functional outcome of inpatient versus outpatient anterior cruciate ligament reconstruction, an equivalence randomized controlled trial with 12 months follow-up.. Knee..

[8] Ng JWG, Smith C, Ilo K, Beavis S, Terry L, Ali F (2019). Dedicated peri-operative pathway improved day case discharge rate for anterior cruciate ligament reconstructions.. Eur J Orthop Surg Traumatol..

[9] Ferrari D, Lopes TJA, França PFA, Azevedo FM, Pappas E (2017). Outpatient versus inpatient anterior cruciate ligament reconstruction: a systematic review with meta-analysis.. Knee..

[10] Stein AL, Baumgard D, Del Rio I, Tutiven JL (2017). Updates in pediatric regional anesthesia and its role in the treatment of acute pain in the ambulatory setting.. Current Pain Headache Rep..

[11] Lefevre N, Klouche S, Doumbouya N, Chambaz M, Devaux C, Thomas R (2014). Hospitalisation en court séjour pour reconstruction du ligament croise antérieur: étude prospective comparative.. J Traumatol du Sport..

[12] Barbier D, N’Dele D, Bennis M, Thevenin-Lemoine C, De Gauzy JS, Accadbled F (2019). Day surgery for anterior cruciate ligament reconstruction in children: a prospective study on feasibility and satisfaction.. J Child Orthop..

[13] Krywulak SA, Mohtadi NGH, Russell ML, Sasyniuk TM (2005). Patient satisfaction with inpatient versus outpatient reconstruction of the anterior cruciate ligament: a randomized clinical trial.. Can J Surg..

[14] Losee GM, Troop RL, Robertson DB, Howard ME Analysis of outpatient ACL surgery: Six year result in 314 patients..

[15] Kao JT, Giangarra CE, Singer G, Martin S (1995). A comparison of outpatient and inpatient anterior cruciate ligament reconstruction surgery.. Arthroscopy..

[16] Kumar A, Bickerstaff DR, Johnson TR, Appleton DF (2001). Day surgery anterior cruciate ligament reconstruction: Sheffield experiences.. Knee..

